# Optimization of the effect of cold plasma treatment on UAE-NADES green extraction of chickpea roots (*Cicer arietinum*) bioactive compounds

**DOI:** 10.1016/j.ultsonch.2025.107276

**Published:** 2025-02-22

**Authors:** Waseem Khalid, Imed E. Benmebarek, Sina Zargarchi, Prashant Kumar, Miral Javed, Andres Moreno, Aanchal Sharma, Gulzar Ahmad Nayik, Tuba Esatbeyoglu

**Affiliations:** a Department of Organic Chemistry, Faculty of Chemical Sciences and Technologies, University of Castilla La Mancha, 13071 Ciudad Real, Spain; b Department of Molecular Food Chemistry and Food Development, Institute of Food and One Health, Gottfried Wilhelm Leibniz University Hannover, Am Kleinen Felde 30, 30167 Hannover, Germany; c Department of Chemical Engineering, Indian Institute of Technology, Tirupati, Yerpedu, India; d School of Engineering, University of Guelph, Guelph, Canada; e University Centre for Research and Development, Chandigarh University, Gharuan, Mohali 140413 Punjab, India; f Marwadi University Research Centre, Department of Microbiology, Marwadi University, Rajkot, Gujarat 360003, India

**Keywords:** Antioxidant activity, Antimicrobial activity, Biomass, Non-thermal treatment, Polyphenol, Ultrasound

## Abstract

The chickpea (*Cicer arietinum* L.) root is an agricultural by-product with the potential for extracting valuable bioactive compounds that often remains underutilized. This study introduces an integrated extraction methodology to enhance the extraction of bioactives using atmospheric air low-pressure cold plasma (CP) treatment followed by ultrasound-assisted extraction (UAE) with natural deep eutectic solvents (NADES). Chickpea root powder was first subjected to CP treatment under optimized conditions (power, pressure, and time) identified via response surface methodology (RSM). Subsequently, UAE-NADES extraction was performed to maximize the results of antioxidant activity (DPPH) and total phenolic content (TPC). The integrated CP-UAE-NADES process enhanced TPC and DPPH compared to the untreated sample (non-CP). The optimum conditions were 11.5  min, 52 W, and 0.65 mbar. The predicted values of the Box-Behnken design for TPC and DPPH were compatible with the experimental Furthermore, microbial load reduction and color stability were analyzed to ensure chickpea root quality and functionality. The combined extraction methodology offers a sustainable and eco-friendly approach for the valorization of chickpea root as a source of bioactives, with potential applications in functional foods, nutraceuticals, and pharmaceuticals.

## 1 Introduction

Chickpea (*Cicer arietinum* L.) is an ancient self-pollinating legume that is believed to have originated in south-eastern Turkey and the neighboring regions of Syria [1]. Chickpea is an annual plant belonging to the Fabaceae, and it grows in semiarid and temperate regions. It is one of the earliest domesticated crops, is globally cultivated, and holds significant importance in the diets of millions [2], [3]. Global chickpea production reaches over 17 million hectares across more than 40 nations primarily located in Asia and the Mediterranean region as well as parts of the Americas. Chickpea ranks as the third largest legume crop globally based on annual yields which surpass 12 million metric tons and India produces the bulk of this global output. Chickpea is cultivated in more than 40 countries, primarily in Asia, the Mediterranean, and parts of the Americas, covering over 17 million hectares globally. It is the third most-produced legume after dry beans, with an annual yield exceeding 12 million metric tons, the majority of which is produced in India [4]. Chickpea serves as a dietary staple due to its rich nutritional profile and versatility in food applications. It is consumed in various forms including whole seeds, flours, and processed products like hummus, falafel, and snacks [5].

Most research has focused on legumes' edible parts [6], [7]. However, legumes are rich in secondary metabolites such as polyphenols, terpenoids, and other bioactive compounds, which are otherwise overlooked and could be further explored [8], [9]. Chickpeas are a good source of bioactive compounds including antioxidants and total phenolic compounds. Moreover, different parts of chickpea such as sprout and root, are rich in bioactive compounds [10]. In the present study, we shift the focus to the roots of chickpea (*Cicer arietinum* L.), a byproduct of the human diet, rich in polyphenols, flavonoids, and antioxidants. These compounds have been described as anti-inflammatory, antimicrobial, and anticarcinogen [11], [12]. The valorization of chickpea roots reducing food waste and adopting sustainable practices. Due to their role in neutralizing free radicals and reducing oxidative stress- which are precursors to certain chronic diseases such as cardiovascular diseases and cancer [9], these compounds offer essential health benefits. With rising consumer demand for natural, health-promoting ingredients, advanced and sustainable extraction methods are required to unleash the full potential of these bioactive compounds [13].

Conventional extraction techniques, such as Soxhlet extraction and maceration, have long been employed to extract bioactive compounds from plant materials. However, these techniques involve using organic solvents such as methanol, ethanol, and acetone, which pose environmental and health hazards. In addition, conventional methods are often time-consuming, energy-intensive, and yield extracts of low purity [14]. To address these limitations, research has increasingly focused on green and sustainable extraction technologies that improve efficiency while reducing environmental impact. Ultrasound-assisted extraction (UAE) with natural deep eutectic solvents (NADES) [10], have emerged as recent approaches.

UAE is a novel extraction technique that leverages ultrasonic waves to enhance the release of bioactive compounds from plant matrices. The use of ultrasonic cavitation mechanically impacts the cell walls. It improves the solvent penetration and accelerates the mass transfer rate, resulting in higher yields and shorter extraction times than traditional methods. UAE operates at relatively low temperatures, making it ideal for preserving heat-sensitive compounds, including flavonoids, phenolics, and antioxidants. Moreover, UAE compatibility with various solvents, including water and green natural solvents makes it a versatile and sustainable option for bioactive compound extraction [15], [16]. NADES represent a revolutionary class of green solvents composed of natural, biodegradable, and non-toxic components such as sugars, organic acids, and alcohols [17]. These solvents emulate the intracellular environment of plants, offering excellent solubility for bioactive compounds while maintaining their stability and bioavailability. NADES are highly customizable; their physicochemical properties can be tailored by adjusting the ratios of their constituents. For instance, a mixture of glycerol, citric acid, and water has proven highly effective in extracting phenolic compounds such as flavonoids from plant materials [18]. By replacing traditional organic solvents with NADES, researchers can reduce the environmental footprint of extraction processes and adhere to green chemistry principles.

CP is a novel, non-thermal technique that produces reactive species, such as electrons, ions, and radicals, to alter the surface properties of plant materials [19]. CP treatment disrupts cell walls and enhances the permeability of cellular membranes, facilitating the release of bioactive compounds during subsequent extraction. In contrast to thermal methods, CP preserves the quality of heat-sensitive compounds, making it particularly suitable for extracting antioxidants and phenolics. Recent advancements have made the system even more effective due to incorporating rotary chambers in the CP system [20].

Based on the current research, it can be deduced that chickpea roots can be considered as an untapped source of valuable bioactive compounds. The combination of CP pre-treatment with UAE-NADES is advantageous in the extraction of the bioactive. Essentially, CP treatment depolymerizes the cell walls and hence increases the extractability of phenolic compounds in the process, enhancing the effects of UAE since it also solubilizes the phenolic compounds. This enhances the extraction yield and supports more environmentally friendly extraction methods, using non-malignant solvents and decreasing energy usage. The present research aims to investigate the combined efficiency of CP, UAE, and NADES for the extraction of bioactive compounds from chickpea roots in terms of total phenolic content, antioxidant activity, color parameters, and microbial load.

## 2 Materials and methods

### 2.1 Collection and preparation of raw materials

Chickpea grains (*Cicer arietinum* L.) were procured from NORMA, Stadtfriedhof Lah supermarket (Hannover, Germany). The grains were washed thoroughly under tap water to remove impurities and soaked in water for 8–10 h to allow for hydration. The hydrated grains were then transferred to sterilized plastic cartridges and kept at 25 °C under controlled conditions to promote root growth. The chickpea cartridges were washed daily, and the chickpea roots were allowed to grow for 8 days, ensuring their complete development. At the end of this period, the matured roots were harvested and thoroughly washed to remove any residual debris. The harvested roots were freeze-dried for 48  h. The dried roots were ground into a fine powder using a laboratory mill and stored in airtight containers at 4 °C until further analysis.

### 2.2 Chemicals

All chemicals and reagents used in this experiment were of analytical grade to ensure the reliability of the analyses. Ethanol (99 %), methanol (99 %), sodium carbonate, and aluminium chloride, gallic acid, 2,2-diphenyl-1-picrylhydrazyl (DPPH) radical, Folin-Ciocalteu reagent, choline chloride, citric acid were purchased from Sigma (Steinheim, Germany). Ultrapure water was prepared with the Purelab® flex 3 from Elga Veolia (Celle, Germany) which was used for all sample preparations.

### 2.3 Preparation of natural deep eutectic solvents (NADES)

NADES were prepared using the method of Koraqi et al. [21]**.** In brief, choline chloride, and citric acid are mixed according to a specific molar ratio. It is then magnetically stirred and heated at 80 °C until a clear homogeneous liquid is formed. The NADES was prepared with water to decrease the viscosity and stored for one day in a dark place after cooling at room temperature.

### 2.4 Extraction with UAE

The ultrasonic bath was used for the extraction process. The freeze-dried chickpea root (50 mg) was mixed with 3 mL NADES. The mixture was sonicated for 30 min, then centrifuged to separate the supernatant at 4500 rpm for 20 min and filtered with filter paper. The extract was stored at 4 °C. All treatments were done in triplicate.

### 2.5 Cold plasma treatment and setup

Cold plasma (CP) was generated under low-pressure conditions using atmospheric air, utilizing the Femto model device (Diener Electronic, Ebhausen, Germany). The apparatus featured a circular borosilicate glass chamber with 8 electrodes, each 100 mm long, operating at a maximum power of 100 W and a frequency of 13.56 Hz. A notable aspect of the setup was its rotational mechanism, enabling the chamber to spin at 10 rpm. Additionally, a rotary pump (Pfeiffer Vacuum, Asslar, Germany) with a 6 m^3^/h capacity efficiently maintained the required low-pressure environment by evacuating the gas. For treatment, freeze-dried chickpea root powder was placed into 200 mL square glass bottles. The process was carried out at power levels of 10, 50, and 90 W for treatment durations of 1, 10.5, and 20 min under pressures of 0.4, 0.65, and 0.9 bar. All experiments were performed at room temperature (25 ± 2 °C) and repeated in triplicate.

### 2.6 Optimization by response surface methodology (RSM)

The model optimized the independent parameters and determined the highest achievable values for the dependent variables. Power, pressure, and time were selected as available factors, each assigned coded levels (−1, 0, +1). The range and levels of the independent variables are shown in Table 1. These levels corresponded to specific values: 10, 50, and 90 W for power; 0.4, 0.65, and 0.9 mbar for gas flow rate; and 1, 10.5, and 20 min for treatment time. The adopted experimental design led to 16 total runs, which were carried out in triplicate (Table 2). The experimental runs were conducted to examine the effects of these factors, with the data analyzed using ANOVA and visualized through 3D response surface plots generated with Design-Expert software 13 (State Ease, Minneapolis, MN; USA).

**Table 1:** Experimental range and independent factors level for response surface methodology design.

Ind. variable	Symbol	Range and Level		
		−1	0	+1
Power (W)	A	10	50	90
Pressure (mbar)	B	0.4	0.65	0.9
Time (min)	C	1	10.5	20

**Table 2:** Response surface methodology experimental runs and their responses.

**Run**	**Factor A:** Power (W)	**Factor B:** Pressure (mbar)	**Factor C:** Time (min)	**Response 1:** TPC **(**m g GAE/g DW**)**	**Response 2:** DPPH **(**mg TE/g DW**)**
1	10	0.65	1.0	34.33	16.23
2	50	0.4	20	42.58	17.05
3	50	0.65	10.5	44.78	21.38
4	50	0.65	10.5	44.87	21.9
5	90	0.9	10.5	31.82	16.2
6	10	0.9	10.5	33.03	13.12
7	50	0.4	1.0	33.27	12.16
8	90	0.4	10.5	35.2	10.53
9	90	0.65	20	37.87	16.67
10	90	0.65	1.0	32.2	17.41
11	50	0.9	20	33.4	16.23
12	10	0.65	20	35.28	13.4
13	50	0.9	1.0	35.43	23.13
14	50	0.65	10.5	46.04	22.61
15	50	0.65	10.5	45.67	22.56
16	10	0.4	10.5	34.27	10.6

### 2.7 Determination of the total phenolic content (TPC) by Folin-Ciocalteu method

The total phenolic content (TPC) of chickpea root powder was determined using the Folin-Ciocalteu method, as described by Singleton and Rossi, [22] with some modifications. For the analysis, 20 μL of the sample extract was combined with 100 μL of Folin-Ciocalteu reagent and 100 μL of sodium carbonate solution. The mixture was incubated at room temperature for 1 h for color development. The absorbance of the reaction mixture was subsequently measured at 765 nm employing with a plate photometer (Infinite® M200, Tecan, Männedorf, Switzerland). Results were expressed as milligram of gallic acid equivalents per g dry weight (mg GAE/g DW).

### 2.8 Determination of the radical scavenging activity using DPPH assay

The radical scavenging activity of the samples was determined using the DPPH (2,2-diphenyl-1-picrylhydrazyl) radical scavenging assay, as described by Bondet et al. [23] with minor modifications. A DPPH solution (300 μM) was prepared in ethanol (99.5%) and incubating it in the dark for 2 h to stabilize. For the analysis, 100 μL of the sample and 100 μL of DPPH solution were added to 96-well plates. The mixture was incubated in the dark for 30 min to allow the reaction to occur. Absorbance was then measured at 515 nm using a microplate reader (Infinite M200, Tecan). Antioxidant result was expressed as milligram of Trolox equivalents per gram of dry weight (mg TE/g DW).

### 2.9 Color measurement

The color changes in freeze-dried chickpea root powder were analyzed using a colorimetric technique with a CM-5 spectrophotometer (Konica Minolta, Langenhagen, Germany). Calibration was performed using a dark light trap and pure white light. Color parameters were assessed using the CIELab scale; L* represents lightness (ranging from 0 for black to 100 for white), a* denotes the red-green axis (negative values indicate green, while positive values indicate red), and b* corresponds to the blue-yellow axis (negative values represent blue, while positive values represent yellow). Color measurements were performed in triplicate at three distinct positions on each sample under D65 illuminat with a 10° standard observer. The analysis was conducted within a wavelength range of 360–740 nm. For comparative analysis, samples with the highest antioxidant activity after plasma treatment were selected and compared to untreated control samples. The total color difference (ΔE) was calculated using the following equation (1):

(1) $$\Delta E=\sqrt{\left(L^{*}-L_{0}^{*}\right)+\left(a^{*}-a_{0}^{*}\right)+{\left(b^{*}-b_{0}^{*}\right)}^{2}}$$

where L*, a*, and b* represent the chromaticity values of the treated samples, while L_0_*, a_0_*, and b_0_* correspond to the values of the control samples.

### 2.10 Microbiological analysis

The microbial analysis of freeze-dried chickpea root powder was assessed by determining the yeast, mold, and total bacterial count (TBC). For the yeast and mold count, 1 g of the chickpea root sample was diluted 10-fold with peptone saline solution and homogenized. Serial dilutions were prepared, and appropriate dilutions were plated on sterilized yeast extract glucose chloramphenicol agar which was autoclaved at 121 °C for 15 min before adding chloramphenicol. The plates were incubated at 25 °C, and the results were expressed as colony-forming units per gram (CFU/g) of dry weight. For the total bacterial count, 1 g of the sample was diluted 10-fold with sterile saline solution. Serial dilutions were prepared, and 1 mL of the appropriate dilution was pour-plated onto sterilized Plate Count Agar (PCA) plates and mixed uniformly, and allowed to solidify. The plates were then incubated at 30 °C for 48 h, and the bacterial counts were recorded and expressed as log_10_ CFU/g.

### 2.11 Statistical analysis

Data analysis was performed using analysis of variance (ANOVA) to assess the interaction between variables (IBM SPSS Statistics 26.0, SPSS Inc., Chicago, IL, USA). The results of microbial and color analysis were presented as the mean ± standard deviation (SD). All experiments were performed in triplicate.

## 3 Results and Discussion

### 3.1 Effect of CP on TPC

**Fig. 1:**
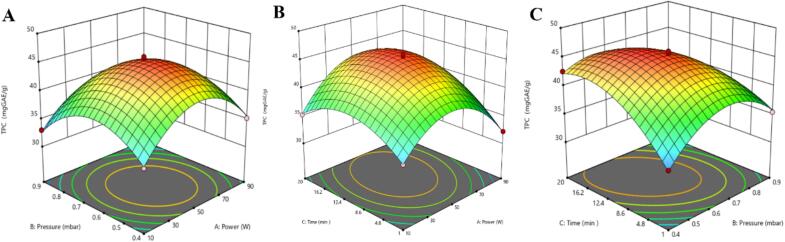
3D response surface methodology: Evaluating the impact of pressure and power (A), treatment time and power (B), and time and pressure (C) on the TPC of chickpea root samples.

To optimize different CP treatment conditions such as power (10–90 W), pressure (0.4–0.9 mbar), and time (1–20 min) were carried out to achieve the highest TPC from chickpea root powder. Table 2 and Fig. 1 present the results that illustrate the interaction between CP treatment parameters and their impact on TPC, ranging from 31.82 to 46.04 mg GAE/g DW across different treatments. It can be seen from Fig. 1A that increasing the power to 50 W and pressure to 0.65 mbar led to a rise in TPC. However, at higher power (90 W) and pressure (0.9 mbar), the TPC result decreased, likely due to the degradation of phenolic compounds. The contour lines emphasize the optimal parameter range, underscoring the balance required to avoid over-processing. Similarly, as shown in Fig. 1B, an increase in the treatment time (10.5 min) and power (50 W), the increase in TPC, and further, an increase in time (20 min) and power (90  W), the TPC result decreased. It can be seen from Fig. 1C that moderate pressure (0.65  mbar) and 10.5 min of treatment resulted in the highest TPC. In contrast, significantly lower (0.4 mbar) or higher (0.9 mbar) pressures and prolonged treatment times led to a significant decrease in TPC. Plasma discharge produces energetic electrons that decompose oxygen molecules into single oxygen atoms, which then react with oxygen molecules to form ozone. It is believed that the molecular ozone degrades phenolic compounds through the rupture of the aromatic ring [24]. These results demonstrate that moderate CP treatment effectively disrupts cell walls through inherent reactive species (such as reactive nitrogen and oxygen species) generated by the plasma, enhancing the extraction and bioavailability of phenolic compounds into the intercellular space [25]. Compared to untreated samples, the highest TPC represented a significant increase, demonstrating the efficacy of optimized CP conditions in enhancing the TPC. In contrast, excessive treatment generates higher reactive oxygen species (ROS) levels, leading to content degradation [20].

These results align with previous studies on the effects of cold plasma treatment on TPC. For instance, Dasan and Boyaci, [26] observed a TPC increase in juice samples after 2 min of plasma treatment, attributing this to reactive species, UV photons, and charged particles, breaking covalent bonds and inducing chemical reactions that enhance cell membrane breakdown. Similarly, Zargarchi and Esatbeyoglu, [20] reported a significant rise in phenolic content in dried ginger treated with cold plasma for 1 min. Similarly, Murakonda and Dwivedi, [14] found that combining atmospheric cold plasma with pulse ultrasound-assisted extraction significantly enhanced the phenolic content of wood apple shells at 15 kV, although higher voltages led to a decline due to surface etching and cell wall rupture. Sarangapani et al. [27] reported a significant increase in phenolic content (*p* < 0.05) at 30 W and 40 W for 5 min, attributing this to the release of phenolic compounds from glycosidic components and the breakdown of larger phenolic compounds into smaller ones. Based on the findings, it can be concluded that the optimal process parameters for extracting the maximum total phenolic compounds from chickpea root powder are a power of 50 W, a pressure of 0.65 mbar, and a treatment time of 10.5 min. These conditions lead to the highest yield of TPC.

### 3.2 Effect of CP on radical scavenging activity

**Fig. 2:**
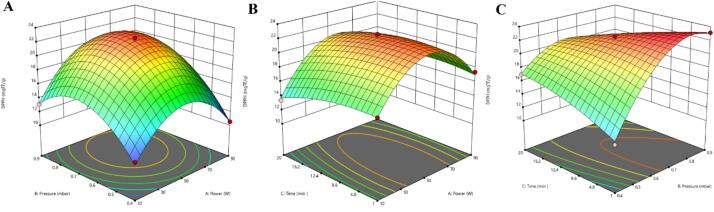
3D response surface methodology: Evaluating the impact of pressure and power (A), treatment time and power (B), and time and pressure (C) on the DPPH activity of chickpea root samples.

Different CP treatment conditions, including power (10–90 W), pressure (0.4–0.9 mbar), and time (10–90 min) were optimized to achieve the highest DPPH activity from chickpea root powder. The results are shown in Table 2 and Fig. 2. Antioxidants play a crucial role in inhibiting free radical activity, stabilizing oxygen, and preventing lipid oxidation, all of which are essential for maintaining food quality and extending shelf life. The DPPH values obtained under different plasma treatment conditions varied from 10.53 to 23.13 mg TE/g DW. As shown in Fig. 2, DPPH activity increased with a slight rise in treatment time and power, pressure, reaching the highest value. However, with further increases in treatment time and power, and pressure, the DPPH activity decreased, demonstrating the efficacy of moderate conditions in enhancing antioxidant capacity. Conversely, the lowest activity occurred under conditions, such as 90 W power, 0.4 mbar pressure, and treatment durations exceeding 10.5 min, likely due to the overproduction of reactive oxygen species (ROS) and reactive nitrogen species (RNS), which degrade antioxidants under excessive plasma exposure. Compared to the untreated, the free radical scavenging activity of atmospheric air plasma-treated samples showed a significant increase. The mechanisms behind the increased antioxidant capacity of CP-treated samples are varied. Reactive species generated during CP treatment, such as superoxide anion (O_2_^–^), hydroxyl radical (OH^•^), and nitric oxide (NO^•^), can break down cell membranes, releasing compounds like polyphenols into the surrounding environment, which boosts antioxidant activity [28]. Murakonda and Dwivedi, [14] also reported enhanced antioxidant capacity in apple wood shell powder, observing a significant improvement when CP treatment was combined with ultrasonic (US) extraction. Bao et al. [29] noted a 30% improvement in the antioxidant activity of tomato pomace extracts after subjecting them to nitrogen plasma treatment, highlighting the role of CP in augmenting bioactive compound availability. Additionally, Tappi et al. [30] an initial increase in antioxidant activity of fresh-cut apples after 10 min of CP treatment could also be due to the oxidation of catechins, which led to the formation of procyanidins that have a higher activity than the former compounds. Nevertheless, extending the treatment time to 120 min resulted in a progressive decrease. However, treatment with a longer time and power reduced DPPH activity. The reduction in the DPPH activity results from the energy electrons emitted during the treatments, which generate single atoms that react with oxygen molecules to form ozone, which is then absorbed by the antioxidant compounds [20]. Hou et al. [31] also noted that the antioxidant activity of blueberry juice declined with the treatment time rising from 2 to 6 min.

### 3.3 Goodness of fit of the model

The analysis of variance (ANOVA) was used to analyze the effects of individual parameters and their interactions on the response variables and evaluate the statistical significance of the models. Statistical parameters, including the *p*-value of the model, Fisher test value (F-value), coefficient of determination (R^2^), adjusted coefficient of determination (R^2^), predicted coefficient of determination (R^2^), coefficient of variation percentage (CV%), and adequate precision, were used to determine the goodness of fit (GOF) for the models. The significance level was considered at 5 % significance (*p* < 0.05). Table 3 shows the statistical parameters of the predicted models, including TPC and DPPH. The results from the predicted models for TPC and DPPH scavenging activity showed low *p-*values, indicating that the models were statistically significant at a 99.99 % confidence level. The F-values, which measure the ratio of model variance to residual variance, were remarkably high for both TPC and DPPH (Table 4), indicating that the models were statistically significant. The probability of the results being influenced by noise was extremely low (*p* < 0.01). The low *p*-values (*p* < 0.0001) further reinforced the accuracy of the models in capturing the experimental data effectively.

**Table 3:** Mathematical formulation that predicts the relationship between the independent variables and the response variable (TPC and DPPH).

**Responses**	**Equations**
TPC	R_1_ = 45.34 + 0.0225 × A − 1.46 × B + 1.74 × C − 0.5350 × AB + 1.18 × AC −2.83 × BC − 6.50 × A^2^ − 5.25 × B^2^ − 3.92 × C^2^
DPPH	R_2_ = 22.11 + 0.9325 × A + 2.29 × B − 0.6975 × C + 0.7875 × AB + 0.5225 × AC − 2.95 × BC − 5.36 × A^2^ − 4.14 × B^2^ − 0.8275 × C^2^

**Table 4:** ANOVA confirmation for DPPH, and TPC responses.

**Responses**	**F-value of model**	***p*-value of model**	**R^2^**	**Adjusted R^2^**	**Predicted R^2^**	**Adequate Precision**	**CV (%)**
TPC	142.01	<0.0001*	0.9953	0.9883	0.9634	30.2552	1.53
DPPH	89.21	<0.0001*	0.9926	0.9815	0.9337	27.7707	3.47

* *p* < 0.05 statistically significant.

The R^2^ values demonstrated a strong correlation between the independent variables and the responses, with TPC achieving an R^2^ of 0.9953 and DPPH an even higher R^2^ of 0.9926 (Table4). These high values indicate that the models explained a significant proportion of the variability in the responses. Additionally, the agreement between predicted R^2^ and adjusted R^2^ values for TPC and DPPH were given in Table 4. The slight difference between these metrics confirms the robustness of the models. The model’s reproducibility was supported by low coefficients of variation (CV%), indicating minimal experimental data scatter and high consistency. Furthermore, the adequacy of precision values exceeded the desirable threshold, confirming a strong signal-to-noise ratio. The experimental validation of predicted values of TPC and DPPH was conducted using confirmation tests based on the experimental design (Table 5). Consistency was observed in the trend between the model’s predictions and experimental results, which closely matched.

**Table 5:** Confirmation tests based on experimental design, comparing predicted and actual results.

Responses	**Predicted Mean**	Predicted Median*	**SD**	**n**	**95 % CI low**	**Data Mean**	**95 % CI high**
TPC	45.37	45.37	0.57	3	44.64	45.70	46.04
DPPH	22.15	22.15	0.58	3	21.39	22.05	22.83

### 3.4 Color

**Fig. 3:**
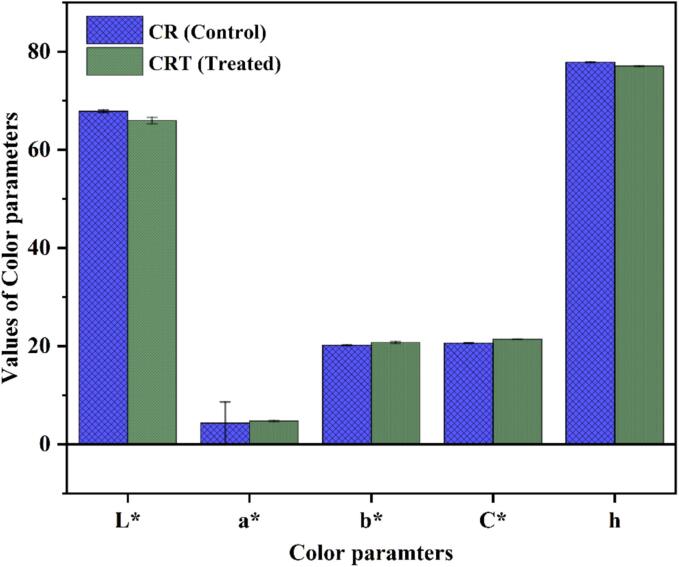
Influence on color parameters of untreated and CP treated chickpea root powder. Values are expressed as mean ± SD (n = 3).

The effect of the CP treated and untreated freeze-dried chickpea powder on color is summarized in Fig. 3. The results showed no measurable changes in the color parameters L*, a*, b*, c*, and h. However, slight variations were observed. The treated sample exhibited a 2.77 % reduction in L* values, suggesting a minor darkening effect potentially caused by oxidation or structural changes in color compounds. The a* and b* values exhibited increases, indicating shifts toward higher redness and yellowness in the treated sample. Furthermore, the chroma value showed an enhancement, reflecting an improvement in color intensity. Additionally, the marginal decrease in h value (0.99%) suggests a minor change in the hue angle, indicating minor changes in the overall hue of the treated sample. Huang et al. [32] stated that the increase in brightness of raisins due to CP treatment was attributed to the plasma-induced denaturation of polyphenol oxidase and peroxidase, enzymes responsible for enzymatic browning in food. Similarly, Hemmati et al. [33] reported that the yellowness and greenness in green tea powder were higher after being subjected to CP treatment, suggesting that this was due to the diffusion of carotenoid and chlorophyll pigments from plasma-damaged cellular structures. In contrast, Beyrer et al. [34] observed a significant decrease in both pigments of powdered *Spirulina* microalgae after atmospheric plasma treatment, attributing the pigment loss to oxidation by generated free radicals. These findings show the necessity of considering consumer preferences, as treatments resulting in lower ΔE values are generally more acceptable. The average color difference (ΔE) in the present study was 2.01, indicating perceptible but minor alterations in the overall color profile. As for information regarding the impact of CP treatment on chickpea root pigments, there is not much literature available. However, evidence from other studies implies that the oxidative species formed during CP treatment may cause oxidation and photo-oxidation. Furthermore, the isomerization of color pigments might also contribute to the observed color changes after CP treatment [20].

### 3.5 Microbial analysis

**Fig. 4:**
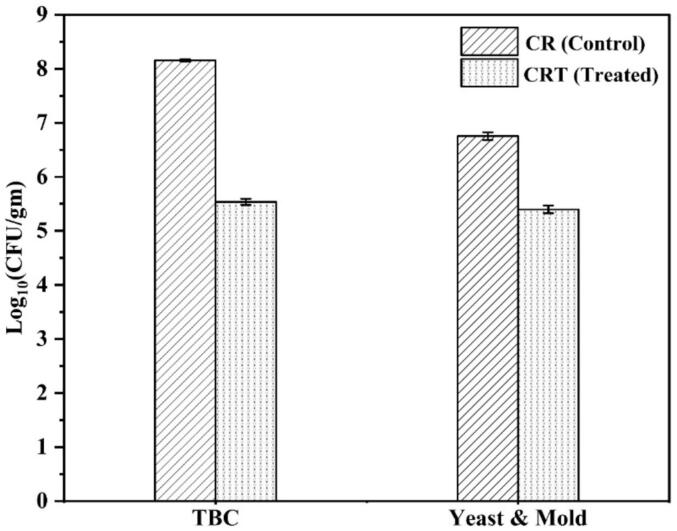
Total Bacterial Count (TBC), yeast, and mold for untreated and CP-treated chickpea root powder. Values are expressed as mean ± SD (n = 3).

The microbial analysis of freeze-dried chickpea root samples, both untreated (CR) and CP-treated (CRT), revealed a reduction in microbial load after treatment as shown in Fig. 4. The TBC showed a decreased 1.40 log reduction, while the yeast and mold counts showed a more minor log reduction of 0.14. These findings highlight the effectiveness of CP treatment in reducing microbial contamination, with a more pronounced impact on bacteria than yeast and mold. Misra et al. [35] observed a 2.40 log reduction in the total aerobic mesophiles and yeast and mold count on strawberries after a 5-minute cold atmospheric plasma treatment. Hemmati et al. [33] observed that CP treatment effectively decreased the total microbial count, mold and yeast, total coliform, *Escherichia coli*, and *Enterococcus faecalis* in green tea. Plasma treatment at 25 kV for 8 min completely inactivated *E. coli* and *E. faecalis* in the sample. Additionally, *E*. *coli* exhibited higher minimum inhibitory and minimum bactericidal concentrations than *Staphylococcus aureus*. The observed microbial reduction can be attributed to the generation of ROS, such as singlet oxygen, oxygen radicals, and hydrogen peroxide, which attack the chemical bonds in the cell membranes of microorganisms. This disruption results in the release of microbial chromosomes, ultimately leading to cell death [33]. Moreover, the reactive species in CP, such as charged particles, free radicals, and UV photons, can break chemical bonds and create lesions (etching) in cell membranes to facilitate further penetration of plasma species into cells. In the case of Gram-negative bacteria, this leads to lysis of their outer membranes and consequent leakage of intracellular materials and cell death [36]. These findings suggest that CP treatment is a non-thermal technique that could improve the quality and safety of chickpea root powder.

## 4 Conclusion

This study explores the potential of combining CP treatment with UAE-NADES to enhance the extraction of bioactive compounds from chickpea root powder. Optimized through RSM, the process achieved superior results with significantly enhanced TPC and antioxidant activity. Moreover, the treatment effectively reduced microbial load and improved color stability, ensuring the quality and safety of chickpea root powder. The synergistic effects of CP and UAE disrupted cellular structures, facilitating higher extraction yields and confirming the efficacy of this green and sustainable method. This innovative approach transforms underutilized agricultural by-products like chickpea roots into valuable functional ingredients for food, nutraceutical, and pharmaceutical applications. The study establishes a foundation for sustainable processing technologies and eco-friendly innovations. At the same time, future research should explore the scalability of this method and the detailed effects of CP treatment on phenolic compounds and antioxidant mechanisms in other food matrices. The optimization of this process could also improve the stability and bioavailability of these compounds, offering a sustainable and green approach to producing high-quality natural extracts for nutraceutical and pharmaceutical purposes.

## 5 Funding acquisition

The publication of this article was funded by the Open Access Fund of Leibniz Universität Hannover.

## CRediT authorship contribution statement

**Waseem Khalid:** Writing – review & editing, Writing – original draft, Software, Investigation, Data curation. **Imed E. Benmebarek:** Writing – review & editing, Visualization, Validation, Methodology, Formal analysis. **Sina Zargarchi:** Methodology, Conceptualization, Writing – review & editing. **Prashant Kumar:** Writing – review & editing, Visualization, Validation, Resources, Formal analysis. **Miral Javed:** Writing – review & editing, Visualization, Validation, Resources, Formal analysis. **Andres Moreno:** Writing – review & editing, Writing – original draft, Visualization, Validation, Supervision, Project administration, Methodology, Investigation, Formal analysis, Data curation, Conceptualization. **Aanchal Sharma:** Writing – review & editing, Software, Data curation. **Gulzar Ahmad Nayik:** Writing – review & editing, Visualization, Resources, Data curation. **Tuba Esatbeyoglu:** Writing – review & editing, Supervision, Resources, Project administration, Methodology, Funding acquisition, Data curation, Conceptualization.

## Declaration of competing interest

The authors declare that they have no known competing financial interests or personal relationships that could have appeared to influence the work reported in this paper.
